# Building evidence-based practice capacity in nursing education: Implementing the academic Best Practice Spotlight Organization^®^ framework

**Published:** 2026-05-15

**Authors:** Theresa Guino-o, Theorose June Bustillo, Freslyn Lim-Saco, Maria Theresa Belciña, Kathleah Caluscusan, Veveca Bustamante, May Ross Café, Mary Nathalie Cata-al, Uenavil Anne Junio, Evalyn Abalos, Rozzano Locsin, Lorraine S. Evangelista

**Affiliations:** 1 Silliman University, Dumaguete, Philippines; 2 Florida Atlantic University, Florida, United States; 3 Sue & Bill Gross School of Nursing, University of California Irvine, 854 Health Sciences Rd, Irvine, California 92617, United States

**Keywords:** evidence-based practice, nursing education, best practice spotlight organization, implementation science, curriculum integration

## Abstract

**Background:**

Translating research findings into healthcare practice remains a major challenge worldwide. The Best Practice Spotlight Organization^®^ (BPSO) initiative, created by the Registered Nurses' Association of Ontario (RNAO), has been broadly adopted in healthcare organizations to promote the systematic implementation of evidence-based practice (EBP) guidelines. In contrast, the use of BPSO principles in nursing education to improve the workforce's readiness for evidence-based healthcare has not been widely studied.

**Objective:**

This study outlines the implementation of an Academic BPSO initiative within a nursing education program in the Philippines.

**Methods:**

We used a descriptive case study to examine how the Academic BPSO framework was integrated into a nursing school. Data were obtained from diverse sources, including institutional documents, curricular resources, program evaluations, and records on faculty development and student participation.

**Results:**

Findings indicate that the Academic BPSO initiative's implementation fostered the incorporation of Evidence-Based Practice (EBP) principles within the nursing curriculum. The initiative succeeded due to institutional support, champion training, and its integration into the curriculum. At the same time, faculty-student interactions promoted learning grounded in guidelines and the practical application of evidence-based care.

**Conclusions:**

Academic BPSO initiatives demonstrate how nursing education programs can contribute to building workforce capacity for evidence-based healthcare by preparing graduates to participate in EBP implementation within healthcare systems.

## 1. Introduction

Despite significant advancements in evidence-based healthcare over the past few decades, integrating research findings into everyday clinical practice remains a global challenge. Evidence suggests that the consistent implementation of proven interventions in healthcare settings often faces delays, thereby hindering the delivery of numerous beneficial practices to patients who would benefit from them [1–3]. Consequently, implementation science has emerged as a vital field of inquiry, focusing on the effective application of evidence-based practices (EBPs) in healthcare. This field underscores the importance of contextual factors, including leadership support, organizational preparedness, and workforce involvement [4,5].

In nursing, the promotion of EBP has been strongly shaped by the Best Practice Spotlight Organization^®^ (BPSO) program created by the Registered Nurses' Association of Ontario (RNAO). The BPSO program was designed to support the systematic implementation of nursing best-practice guidelines through organized collaborations between healthcare providers and academic institutions [6]. Participating organizations pledge to a multi-year endeavor to implement, assess, and sustain these principles, supported by governance frameworks, leadership engagement, and the establishment of networks of skilled advocates for best practices [7]. This structured approach aims to help translate research evidence into clinical practice and encourage lasting organizational change in healthcare delivery [6,7].

International studies show that the BPSO framework can help organizations adopt EBPs and foster a culture that supports ongoing quality improvements [6–9]. Evaluations of BPSO implementation within healthcare systems, for instance, have pinpointed crucial success factors, such as executive backing, strong governance frameworks, and dedicated champion networks that facilitate engagement among clinical teams [7,9]. Simultaneously, investigations into the experiences of nurses involved in BPSO initiatives underscore the importance of institutional support, structured training, and dedicated time for implementation in maintaining EBP initiatives [8].

Central to the BPSO model is the role of trained champions, who act as knowledge brokers within their organizations, facilitating the dissemination and application of best-practice guidelines and supporting colleagues in integrating research evidence into clinical decision-making [6]. Champions often gain from well-structured training programs and collaborative, interdisciplinary teams. These teams are responsible for overseeing the adaptation, implementation, and evaluation of the prescribed guidelines [7]. The collaborative framework, a key part of the BPSO model, aims to connect knowledge creation with its practical use in healthcare. This is done by creating environments that emphasize continuous learning and the use of evidence-based methods [9].

Although the BPSO program has been widely implemented in healthcare settings worldwide, its fundamental tenets have received comparatively less attention in integrating into academic nursing programs, which are crucial for preparing future clinicians to engage in EBP. Nursing education plays a crucial role in developing the skills, attitudes, and leadership qualities needed to effectively turn research findings into practical applications [10–12]. Therefore, incorporating BPSO-informed strategies into nursing programs could be a vital means of strengthening the link between academic training and the practical application of evidence-based care in clinical settings.

**Figure 1.**
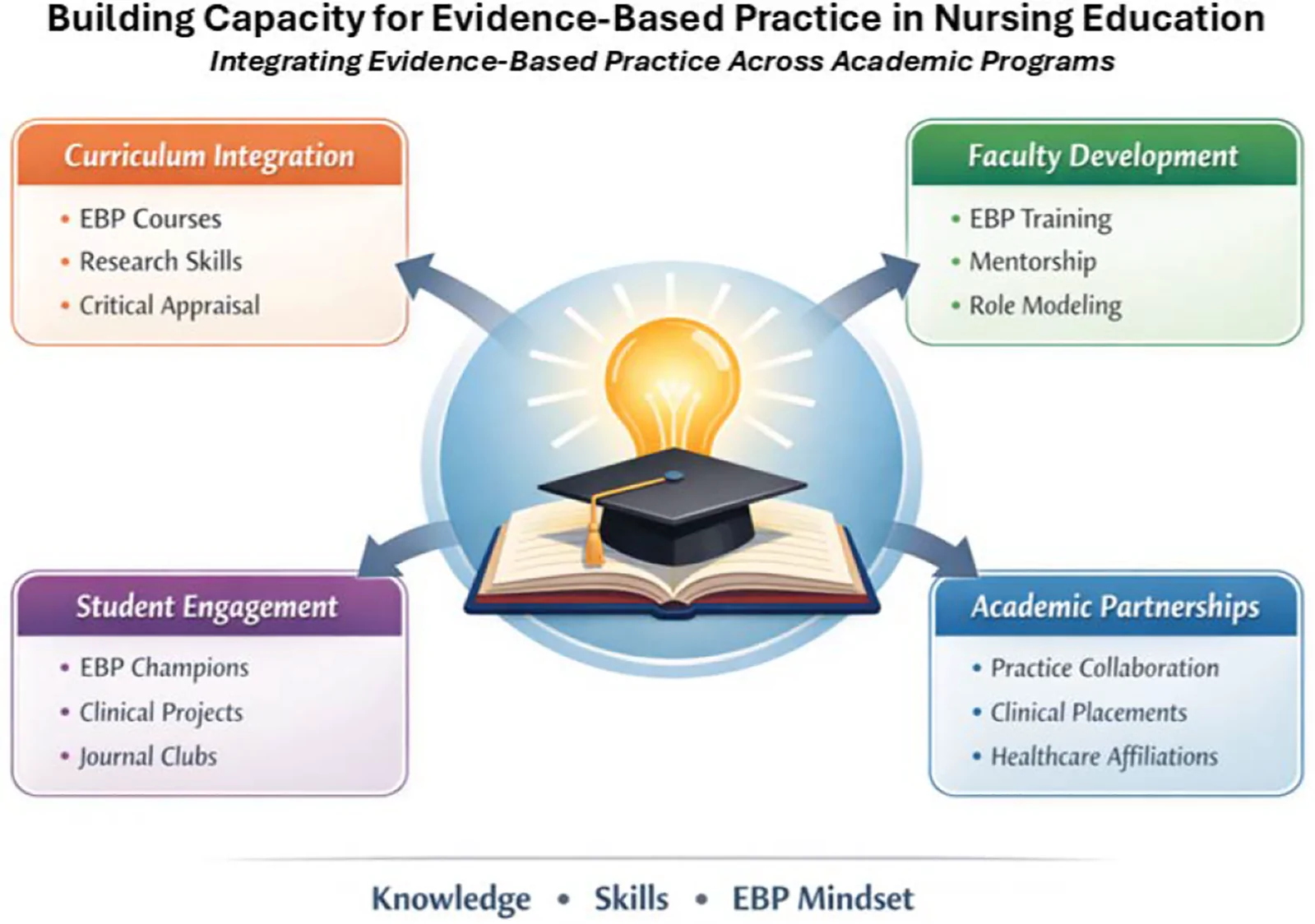
The figure presents a conceptual framework, illustrating how nursing education programs can cultivate evidence-based practice competencies through curriculum integration, faculty development, student engagement, and academic-clinical partnerships. These interconnected elements contribute to the development of graduates who possess the knowledge, skills, and professional mindset necessary to implement and sustain evidence-based practice within healthcare systems

The purpose of this paper is to describe the implementation of an academic BPSO initiative within a nursing education program and to examine how institutional commitment, champion training, curriculum integration, and student engagement strategies can support the development of a workforce prepared to apply EBP in clinical settings. To conceptualize how academic institutions can operationalize the principles of the BPSO framework within nursing education, this initiative adopts a capacity-building perspective that highlights the institutional processes required to prepare graduates capable of implementing EBP in clinical settings (Figure 1).

## 2. Conceptual framework

The Academic BPSO initiative, created by the RNAO, serves as a prominent global strategy for systematically integrating nursing best-practice guidelines. This program promotes structured guideline implementation through organizational commitment, leadership engagement, faculty and student champion training, and ongoing outcome assessment [7]. Studies in healthcare settings have shown that these approaches can improve nurses' commitment to EBP. They also help create organizational cultures that support lasting changes in practice [8,9]. Skilled champions act as vital knowledge intermediaries. Faculty-student and student-student academic engagements are multiplier opportunities that expand the reach, efficiency, and impact of best practices across hospital and community clinical sites.

Thus, the BPSO framework in healthcare provides a robust basis for improving EBP competencies in nursing education. A comprehensive approach is crucial for preparing graduates to use EBP effectively, rather than just offering separate courses on research methods or critical evaluation. An integrated educational strategy is essential. This strategy requires support from the institution, faculty, curriculum design, and practical learning experiences to develop the skills needed for evidence-informed clinical practice.

Figure 1 presents the conceptual framework for enhancing EBP skills in nursing education through academic BPSOs. The model shows that institutional commitment is essential for driving change, thereby supporting the development of champion training programs. These programs aim to give faculty and students the tools to spearhead EBP projects. These individuals, in turn, help weave evidence-based guidelines into course content and learning experiences. This helps students engage with EBP concepts. As a result, students can apply these skills in real-world settings, such as clinical internships and other hands-on learning experiences.

The academic BPSO implementation pathway aims to prepare graduates to contribute to evidence-based healthcare systems by moving from institutional commitment to clinical application. By integrating EBP concepts into nursing education and aligning them with clinical implementation strategies, academic institutions can significantly enhance the workforce capacity needed to sustain EBP practice in healthcare

## 3. Materials and methods

### 3.1 Design:

This initiative employed a descriptive implementation case study design to examine the integration of the BPSO framework within an academic nursing program. Case study approaches are frequently used in implementation research to document how organizational strategies, leadership processes, and contextual factors contribute to the adoption and sustainability of EBPs in real-world settings [2]. This paper outlines the steps a nursing school took to implement the Academic BPSO initiative and integrate EBP principles into the educational setting. The project aimed to document how the institution incorporated best practice guidelines into its curriculum. It also examined how faculty and students became advocates for these practices and how they were involved in learning activities supported by evidence.

### 3.2 Setting:

This project was conducted at the School of Nursing at Silliman University, Philippines, which participates in the RNAO-BPSO program. Within the framework of the Academic BPSO initiative, the school dedicated itself to integrating RNAO Best Practice Guidelines into its curriculum and fostering a learning environment conducive to EBP. Joining the Academic BPSO program means working alongside healthcare partners. It also means participating in a structured implementation process. This process includes securing leadership support, developing champions, incorporating guidelines into educational programs, and tracking implementation outcomes. These activities aim to strengthen nursing graduates' preparation to participate in EBP initiatives in clinical settings.

### 3.3 Data sources:

The data underpinning this initiative were derived from a variety of sources on the Academic BPSO initiative within the nursing program. These encompassed institutional planning documents, implementation reports, curricular resources, training records for BPSO champions, and records of student engagement in activities aligned with the RNAO Best Practice Guidelines. Furthermore, program reports detailing how best-practice guidelines were incorporated into course content, student learning activities, and clinical practice experiences were examined. Together, these sources highlighted the strategies used to promote the application of the Academic BPSO framework and develop EBP skills among nursing students.

### 3.4 Analytical approach:

**Figure 2.**
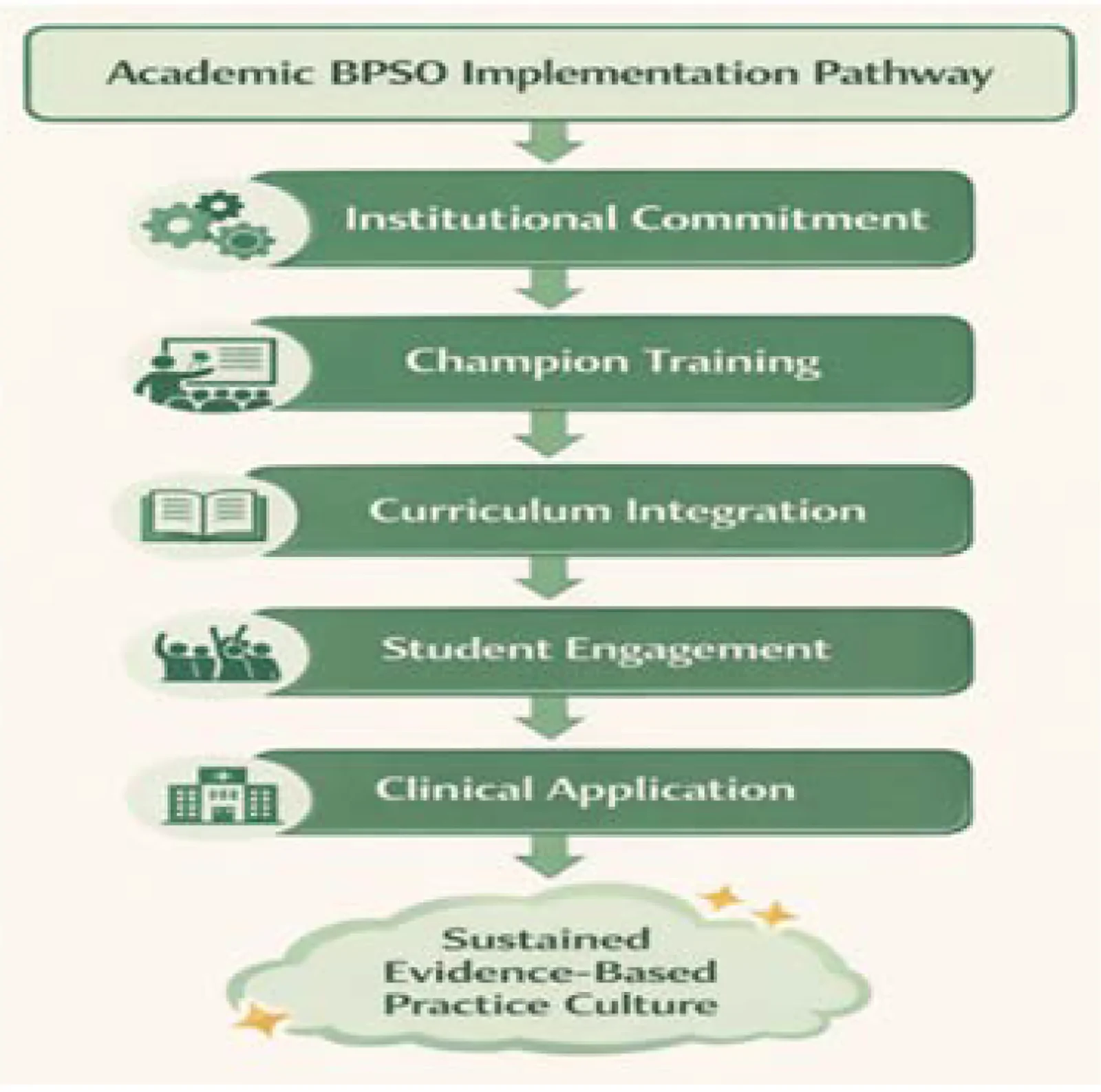
Academic BPSO Implementation Pathway for Integrating Evidence-Based Practice in Nursing Education. Illustration of the phased process used to embed evidence-based practice within a nursing program through institutional commitment, champion training, curriculum integration, and student engagement, culminating in clinical application and the development of a sustained evidence-based practice culture

A descriptive analytical method was used to evaluate the implementation of the Academic BPSO initiative within the nursing program. Data were analyzed and organized to identify the main processes and strategies that facilitated the integration of EBP in the academic environment. The analysis identified key factors, including institutional conditions, leadership, educational strategies, and activities that encouraged the adoption of best-practice guidelines. The findings were organized according to the stages of the Academic BPSO implementation process illustrated in Figure 2, including institutional commitment, champion training, curriculum integration, student involvement, and clinical practice. This approach demonstrated how the BPSO framework's principles were applied in an academic environment and how these strategies facilitated the development of EBP skills in nursing education.

### 3.5 Ethical considerations:

This paper describes a program implementation initiative without identifiable participant data and was therefore exempt from institutional ethics review.

### 3.6 Reporting considerations:

This initiative describes the implementation of an academic BPSO effort within a nursing education curriculum. The principles of implementation science guided the outline of program activities, institutional strategies, and observable outcomes. These principles highlight the importance of documenting contextual factors, procedures, and capacity-building strategies that support the integration of EBPs into educational and healthcare systems [1–3].

## 4. Results

The Academic BPSO initiative was progressively integrated into the nursing curriculum to enhance EBP competencies within the educational setting. This process, as shown in Figure 2, included several interconnected stages: Obtaining institutional support, training key personnel, integrating the curriculum, engaging students, applying EBP in clinical settings, and ultimately building a sustainable culture of EBP.

### 4.1 Institutional commitment:

The implementation of the Academic BPSO initiative in the nursing program depended on the institution's commitment. Silliman University College of Nursing's leadership formally approved joining the BPSO program, aligning it with the university's overall strategic aims for academic achievement, research integration, and enhancing healthcare education quality. Leadership involvement was crucial in creating a shared vision for evidence-based practice within the academic setting. Faculty were prompted to incorporate best practice guidelines into their pedagogical approaches and to facilitate students' participation in evidence-informed learning opportunities. Consequently, institutional leadership helped create a conducive environment for integrating EBP principles into the nursing curriculum across classroom and clinical experiences.

### 4.2 Champion training:

A crucial element of the Academic BPSO implementation was cultivating faculty and student champions. These individuals led and supported the integration of EBP into the educational setting. As program champions, they served as essential experts, actively sharing guideline-related information with both faculty and students. They provided guidance, helped shape course content, and championed the integration of EBPs across all classes, clinical training, advocacy programs, and even extracurricular activities. All faculty members were trained as pioneer champions centered on the RNAO Best Practice Guidelines, with a specific emphasis on promoting evidence-based decision-making within clinical and educational environments.

### 4.3 Curriculum integration:

A central approach to enhancing students' abilities to utilize research evidence in clinical judgment involved integrating EBP principles into the nursing curriculum. Course coordinators and faculty champions collaborated to identify ways to incorporate RNAO Best Practice Guidelines and other evidence-based resources into the current course offerings.

Curriculum integration necessitated incorporating EBP principles into classroom teaching, skills practice, simulation experiences, clinical case analyses, and student assessments, all of which prioritized the critical evaluation of research findings. The overarching objective of these pedagogical strategies was to deepen students' understanding of the impact of research outcomes on clinical decision-making while simultaneously cultivating their capacity to assess and implement optimal practice guidelines in patient care.

### 4.4 Student engagement:

A key component of the Academic BPSO initiative was student engagement. Students were initially introduced to the fundamental tenets of EBP. Subsequently, they engaged in exercises intended to facilitate the application of evidence within clinical environments. The core methodologies comprised journal discussions, analysis of best-practice guidelines, and engagement in evidence-based clinical projects. These activities helped students improve skills in evaluating evidence, understanding guidelines, and working together effectively to solve problems in clinical settings.

### 4.5 Clinical application:

Students applied their clinical experiences to implement EBP principles in real healthcare environments, specifically at the base hospital, Silliman University Medical Center, and the field laboratories in the community. Through engagement with clinical institutions participating in BPSO initiatives or using evidence-based guidelines, students had the opportunity to observe and actively contribute to implementation activities in these practical contexts. These experiences reinforced what was learned in class. They helped students see how guidelines are applied, observe teamwork across different fields, and consider how EBPs influence patient care. As a result, clinical learning environments become a vital link between academic training and professional practice.

### 4.6 Sustained EBP culture:

Throughout its duration, the Academic BPSO initiative strengthened the culture of EBP within the nursing program. Faculty members showed greater engagement with best-practice guidelines, and evidence-based concepts were more consistently integrated into both coursework and clinical settings. As a result, graduates developed a deeper understanding of evidence-based concepts and gained clearer insight into how clinical guidelines improve healthcare quality and patient outcomes. The effort supported the development of a workforce capable of implementing and sustaining evidence-based healthcare practices by incorporating EBP into educational programs and clinical training. Many of these graduates notably transitioned into hospital roles, allowing them to continue championing evidence-based excellence in clinical practice.

## 5. Case illustration: Academic BPSO implementation in nursing education

This case study illustrates how the Academic BPSO initiative was implemented within the nursing program. It examines how an RNAO Best Practice Guideline was incorporated into both the curriculum and the clinical learning environment. This example highlights how institutional commitment, strong leadership, curriculum integration, and student involvement work together to successfully implement EBPs in a real-world setting.

### 5.1 Institutional commitment:

The School of Nursing's leadership was a significant source of support for the Academic BPSO program. The integration of EBP into the curriculum was encouraged, and faculty received assistance in implementing established standards. The institution's support facilitated the alignment of the Academic BPSO initiative with overarching program objectives, including quality improvement, research engagement, and the preparation of graduates for evidence-based healthcare settings.

A soft launch of the program was held, during which school administrators, clinical partners, and Local Government Unit officials shared their understanding and commitment to the healthcare initiative. Essential resources were allocated for program implementation, with the involvement of various stakeholders. This included faculty development initiatives, training programs for champions, curriculum assessments, and support for health institutions.

### 5.2 Champion leadership and curriculum integration:

The integration of BPSO principles into the nursing program relied on faculty leadership actively promoting the initiative. They collaborated with course coordinators to incorporate specific RNAO Best Practice Guidelines on breastfeeding, vascular access devices, and pressure injuries into the course structure. This initiative included lectures, class discussions, and student assignments.

The guidelines found their way into clinical decision-making lectures. Students then engaged in activities, including examining supporting evidence and considering how the guidelines might be applied in actual patient care. Faculty champions led discussions that helped students understand how clinical guidelines are applied in healthcare settings.

Furthermore, student champions were trained in guideline implementation and played a vital role in disseminating information through group discussions and social media platforms. To sustain the number of student champions after graduation turnovers, additional students were encouraged to participate in subsequent trainings offered on-site or online in the BPSO program.

### 5.3 Student engagement and clinical application:

Students engage with EBP principles through both academic coursework and clinical experiences. Their education encompasses the critical evaluation of research findings, the examination of guideline recommendations, and the investigation of how evidence-based interventions can improve patient outcomes. These hands-on experiences bridge the gap between theoretical understanding and clinical application, thereby deepening students' comprehension of the effects of guideline-driven care on patient outcomes.

Beyond clinical applications, students used the BPGs to fuel their advocacy programs. They also engaged in information drives, exhibits, and fun activities to enhance knowledge reach to other healthcare students. The students co-organized and presented at international conferences within academic BPSO circles.

### 5.4 Outcomes and sustained change:

Including the academic BPSO initiatives in the nursing curriculum enhanced faculty and student understanding of EBP. Faculty members noticed an increase in the use of guideline resources and a more integrated application of evidence-based principles. Students showed a stronger understanding of clinical guidelines and expressed greater confidence in their ability to provide evidence-based care. As a result, these initiatives fostered a strong culture of evidence utilization within the nursing program. This cultural shift created a ripple effect: as faculty improved their teaching methods and students advocated for best practices in clinical settings, the influence of BPSO guidelines extended beyond the original curriculum. The Academic BPSO initiative effectively connected academic learning with real-world clinical practice. As a result, graduates were better equipped to engage in evidence-based projects within healthcare organizations, acting as change agents who promote high-quality care standards across the broader health system.

## 6. Discussion

This paper explains how Academic BPSO programs can enhance the integration of evidence-based practice in nursing education by ensuring institutional support, leadership endorsement, curriculum inclusion, and active student participation. In low- to middle-income countries, this initiative is a crucial approach for strengthening health systems by training nurses to enhance clinical outcomes despite limited resources. By developing “change agents” within the classroom, the BPSO model allows graduates to promote cost-efficiency. These evidence-based interventions reduce medical errors and enhance patient safety in resource-constrained settings.

The successful implementation of EBP programs in academic settings depended on strong institutional commitment. The Academic BPSO initiative's alignment with institutional goals, backed by leadership support, promoted faculty involvement and secured resources. For example, adopting the best practice guideline on breastfeeding highlights the importance of providing thorough education to mothers and families on techniques such as correct latch and positioning.

This initiative aimed to address gaps in health education methods, resolve discrepancies in instructional materials used by students, and enhance prenatal breastfeeding education for young mothers. Although maintaining standardized educational resources remains difficult in low- and middle-income countries like the Philippines, student and faculty champions facilitated access to essential materials and incorporated breastfeeding education into routine clinical learning despite resource limitations.

The findings support existing implementation science research, which emphasizes that organizational readiness, leadership support, and an appropriate infrastructure are crucial for successfully implementing evidence-based interventions [1,3]. Furthermore, research on the implementation of BPSO programs in healthcare settings has identified analogous determinants, suggesting that leadership involvement and established governance structures promote the uptake of best-practice recommendations [7].

Faculty champions were crucial in implementing activities within the academic setting. Champions acted as knowledge brokers by promoting guideline recommendations and encouraging faculty and students to engage in EBP. This role supports prior research emphasizing the significance of champion networks in BPSO programs for enhancing knowledge translation and driving practice change [8,9]. Champions can also foster mentorship and collaborative decision-making within the routine of education.

Curriculum integration was a crucial method for strengthening EBP skills in nursing students. The Academic BPSO initiative encouraged integrating guideline recommendations and evidence appraisal activities throughout the curriculum, instead of confining EBP instruction to separate research courses. This approach aligns with nursing education research, which emphasizes the importance of incorporating EBP principles into academic programs. This integration aims to improve critical thinking, clinical judgment, and evidence-based decision-making skills [10].

Student engagement activities enhanced these learning experiences, enabling students to actively use evidence-based concepts in classroom discussions and clinical settings. Exposure to EBP initiatives and participation in guideline-based learning activities in healthcare environments facilitate students' understanding of the significance of research evidence in clinical decision-making. These experiences are essential for bridging the gap between academic learning and clinical practice, a significant obstacle in the implementation of evidence-based healthcare.

The findings of this initiative indicate that academic institutions play a significant role as upstream contributors to the development of healthcare systems that can sustain EBP. Integrating BPSO principles into nursing education prepares academic programs to develop graduates skilled in evidence-based guidelines and capable of implementing strategies in clinical settings. As a result, academic BPSO programs can strengthen the link between nursing education and healthcare practice, helping build a workforce ready to deliver evidence-based care for better health outcomes.

## Conclusion

The Academic BPSO initiative shows how nursing education programs can enhance workforce capacity for evidence-based healthcare. Institutional commitment, leadership champions, and curriculum integration support ongoing EBP adoption despite resource limitations. These findings highlight the value of academic–clinical alignment in preparing graduates to support EBP across healthcare systems.

## Future directions

Future endeavors should focus on expanding Academic BPSO initiatives across nursing education to improve the integration of EBP into academic curricula. This effort requires stronger collaboration between academic institutions and clinical settings that actively use EBP, thereby reinforcing the link between educational theory and practical application. Consistently integrating best-practice guidelines into the nursing curriculum, along with continuous mentorship and capacity-building efforts for both faculty and student leaders, is essential for maintaining these initiatives. Regular assessments of academic–clinical collaborations are essential for identifying effective strategies to incorporate guideline implementation into student training. This process prepares graduates for leadership roles in healthcare improvement.

## Data Availability

No new data were generated or analyzed in this study.

## Highlights

- Academic Best Practice Spotlight Organization^®^ (BPSO) integrated evidence-based practice (EBP) throughout the entire curriculum.
- Institutional leaders supported champion training and curriculum updates.
- Faculty-student engagement improved guideline-based learning.
- Graduates demonstrated improved preparedness for implementing EBP.
- Academic BPSOs act as bridges between research evidence and clinical application.

## Abbreviations

The following abbreviations are used in this manuscript:

BPSO: Best Practice Spotlight Organization
EBP: Evidence-Based Practice
RNAO: Registered Nurses' Association of Ontario
